# Cumulative inhibition in neural networks

**DOI:** 10.1007/s10339-018-0888-z

**Published:** 2018-11-03

**Authors:** Trond A. Tjøstheim, Christian Balkenius

**Affiliations:** Lund University Cognitive Science, Lund University, Box 117, 221 00 Lund, Sweden

**Keywords:** Cumulative inhibition, Multi-resolution, Coarse-to-fine processing, Unsupervised learning, Acuity, Cortical microcolumn, Visual cortex

## Abstract

We show how a multi-resolution network can model the development of acuity and coarse-to-fine processing in the mammalian visual cortex. The network adapts to input statistics in an unsupervised manner, and learns a coarse-to-fine representation by using cumulative inhibition of nodes within a network layer. We show that a system of such layers can represent input by hierarchically composing larger parts from smaller components. It can also model aspects of top-down processes, such as image regeneration.

## Introduction

In a dynamical environment, it is essential for animals to react quickly, but they also need to finely discriminate between stimuli, like those associated with food and mates. To accommodate these dual requirements, perceptual networks in mammals represent information in both a coarse and a finely detailed manner. Several studies have suggested that cortical development is closely tied to the formation of these abilities (Hughes 1977; Boothe et al. 1985; Fagiolini et al. 1994; Huang et al. 1999; Prusky and Douglas 2003).

So far, biologically plausible models of such combined representations in artificial networks have been scarce. Models capable of unsupervised learning have been particularly elusive. In this paper, we attempt to address the issue of unsupervised learning of coarse-to-fine representation, and how it can be implemented in an artificial system.

A notable feature of mammalian cortical development is the presence of critical periods (Hensch 2005). During such periods, the cortex displays heightened plasticity, and it is thought that this reflects perceptual processes that adapt to the fundamental statistics of the sensorium (Freeman and Marg 1975; Boothe et al. 1985; Bao 2015). Once these self-organizing processes have stabilized, plasticity decreases (Hensch 2005).

Interpreting critical periods as adaption would predict a longer critical period for more variable environments, as well as for a more complex sensory apparatus. This is indeed what has been found (Hensch 2005), with plasticity windows ranging from around a week for mice (Huang et al. 1999), up to several years for humans (Hensch 2005).

During the critical period, perception of detail, or acuity, increases (Huang et al. 1999). This increase is progressive and develops over time. Apart from the optics of the eye, visual acuity depends primarily on the density of retinal ganglion cells, and secondly on the interpretative ability of the visual cortex (Hughes 1977; Huang et al. 1999).

The requirement for multi-resolved representation is motivated by a need for both fast pattern recognition and detailed pattern recognition. Multi-resolved representation (Navon 1977; Schyns and Oliva 1994; Pomerantz 1983; De Valois et al. 1982; Ullman et al. 2002; Simoncelli 2003) implies that at least two representations are available: a coarse grained representation and a finer grained one. Acuity measures the highest possible resolution of perceptual representation, and hence, the fine end of the multi-resolved spectrum. Whether more than two levels of detail is required or present in biological systems is a subject for further investigation.

Visual acuity increases significantly during the first post-natal year in mammals (Boothe et al. 1985). This development appears to be mediated by increasing innervation of inhibitory inter-neurons in the primary visual cortex (Huang et al. 1999), and is dependent on the activity of pyramidal cell populations that receive signals from retinal ganglion cells (Freeman and Marg 1975; Fagiolini et al. 1994). A similar, but longer term process appears to be involved in the episodic memory system, and further along the occipitotemporal pathway (Keresztes et al. 2017).

As acuity increases, however, the need for rapid detection of ecologically important stimuli remains. Hence it appears reasonable that the brain does not outgrow its low-resolution representations, but rather maintains their approximations to afford quick appraisal. This leads to the concept of multi-resolution, which implies that a system learns and uses representations of differing resolution, depending on the requirements of the situation (Navon 1977; Schyns and Oliva 1994; Pomerantz 1983; De Valois et al. 1982; Ullman et al. 2002; Simoncelli 2003).

For biological systems, one of the most salient uses for multi-resolved representations is in the categorization of stimuli. In this context, coarse representations allow quick reactions in the presence of predators, while finer representations allow an animal to discriminate between what can be eaten and not, which con-specifics are of the opposite sex, or who can be groomed. For artificial neural networks, on the other hand, coarse representations can make for quicker response times and lower energy costs, while finer representations increase classification accuracy.

**Fig. 1:**
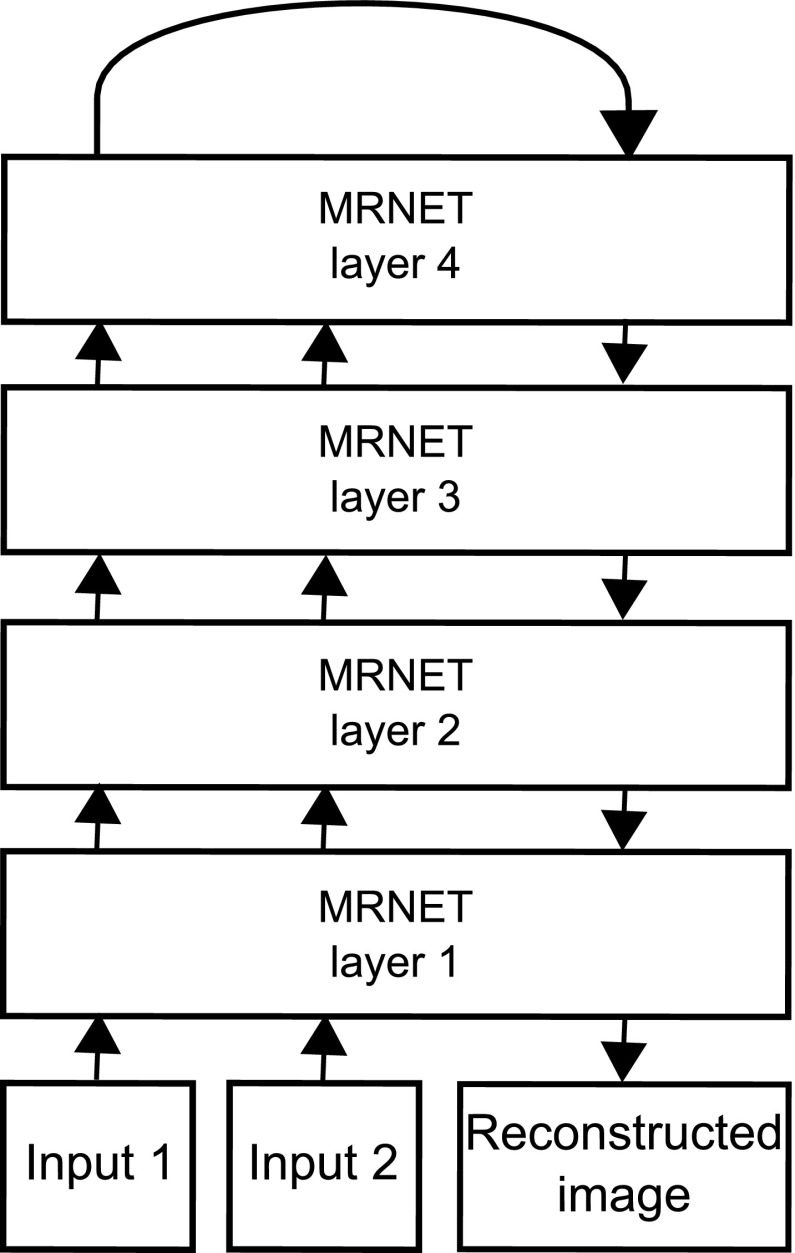
MRNET computational graph. Bottom-up input is a set of 36 × 36 arrays of floating point values, like intensity values from an image. In this particular configuration, two bottom-up inputs are used to be able to show the refinement process on a constant image while learning also from a variable image stream. The input images are fed into layer 1 of the model, where they are transformed into a representation which takes into account level of detail. Weights are updated by iterating over the set of inputs. In addition to the bottom-up input, each layer has also a set of top-down inputs, where the reverse operation is performed: By convolving the layers’ weights with the top-down inputs, images of the same format as the bottom-up input is synthesized. At layer 1, the synthesized output is a 36 × 36 image. In the present model, by connecting the first bottom-up output of layer 4 with the top-down input, input images are reconstructed

Briefly, the model presented here consists of a number of processing layers arranged in a hierarchical structure. The model takes in a gray-scale image, and each layer uses a filter bank that adapts to represent statistical regularities of the input. Using the weight update algorithm detailed below, the filters adopt their representations with varying levels of detail. Layers closer to the input have filters that cover a smaller area of the input, while the top most layer has filters that cover the area of the entire input image. Using linear combinations of the learned filters, the input can be regenerated through top-down processes. See Fig. 1 for a diagram of the model.

The model attempts to map to the biological mechanisms described above, in the following way. The critical period in acuity development corresponds to the training period for the various layers, where layers nearest to the input must necessarily stabilize before layers further up in the hierarchy. This implies that more complex processing that involves more layers takes a longer time to stabilize. The multi-resolved strategies map to our proposed mechanism of cumulative inhibition.

Consequently, the major key points of the model are as follows. Firstly, it learns multi-resolution representations in an unsupervised way. Secondly, it can regenerate arbitrary representations in a top-down fashion. Thirdly, it suggests a possible mechanism for multi-resolution representation in biological networks by means of cumulative inhibition in the sensory cortices.

It is not obvious that a model concerning itself with how acuity develops needs to concern itself with multi-resolved organization. However, as we attempt to show, the proposed cumulative inhibition mechanism appears to tightly intertwine multi-resolution and acuity, such that the latter is an effect of the former. That is, the process of cumulative inhibition forms receptive fields responding to varying degrees of complexity. As these fields develop, they support perception of more detail, and hence higher acuity.

The rest of this paper is structured in the following way. First, a background section will present past approaches to implementing multi-resolution functionality, and different theories of how multi-resolution relates to human perception. Reverse hierarchy theory of multi-resolved representation will then be highlighted and discussed, before anti-Hebbian learning and sparse coding is presented as a possible mechanism for achieving multi-resolution in biological networks. The background section will end with a presentation of the mammalian canonical microcircuit. These cortical networks appear to have in place the machinery to support formation of multi-resolved receptive fields.

Under the method section, we describe the architecture and the experimental setup, along with an overall description of the novel multi-resolution network (MRNET) and its algorithms. This is followed by a short description of how the system was trained. Next, we present the results of the experiment with figures showing how the system adapts to the training input.

Finally, in the discussion, we attempt to compare the measured behavior of the experimental system with current knowledge of how biological neural systems develop. We also address some criticisms against sparse coding, and suggest some ways they might be resolved in the context of cumulative inhibition.

## Background

In this section, we present a selection of past approaches to multi-resolution representation, and theories on how multi-resolution relates to perception. We present the reverse hierarchy theory of multi-resolved representation in particular. Possible mechanisms for such representations are anti-Hebbian learning and sparse coding, which is presented next. Finally, a brief overview of the mammalian canonical microcircuit is given. These circuits are candidate sites for mediating multi-resolved representation in mammalian brains.

### Multi-resolution representation

In the context of coarse-to-fine processing, Witkin (1984) noted that a signal’s extreme values are useful for compactly describing that signal, since they frequently point to edges or other semantically important features. Thus, extremes can be used to produce a coarse sketch of the signal. But it is difficult to determine at which scale to filter events. Using a method called scale-space filtering, Witkin showed how several descriptions of a signal can be produced by convolving it with a Gaussian mask with a range of sigma values. A small sigma value will conserve high frequency information, while a large sigma will smooth out those frequencies, leaving only lower frequency information. The resulting representations were arranged into a tree structure. This structure is called the interval tree, and is constructed by the following process. First, the second derivative is applied to the product of the convolution operation. This yields a set of zero-crossings for each curve. Secondly, these zero-crossings are used to create the so-called undistinguished intervals. Such an interval is bounded by extremal points, but contains no extremal point within it. An undistinguished interval becomes a node in the interval tree. The node can be expanded by considering the curve of the next more detailed sigma value, and adding all the undistinguished intervals that are encompassed within. This process is continued until the curve with the smallest sigma value has been processed. The interval tree hence contains information about the source signal at all scales. But to find the “right” scale, that is the scale which “pops out” to human perception, Witkin considered the stability of bounding extrema across scales. He found that those extrema which were most stable can be used to generate human-salient intervals. Less-stable intervals can then be discarded to simplify and constrain the interval tree.

Another type of multi-resolution strategy is used in one of the most successful object detection algorithms so far (Viola and Jones 2001). A cascade of coarse-to-fine templates is used to detect objects, such as faces, in an image. The input is then analyzed at progressively higher resolution. At each stage, input patterns that do not match adequately are discarded. At the highest resolution, only a few candidates then remain to be analyzed.

For reinforcement learning, similar problems occur for classifying situations and associating them with the correct actions (Sutton 1996). Sutton (1996) employed sparse and coarse coding of function approximators to solve control problems. His approach built on the “cerebellar model arithmetic computer” neural network (Miller et al. 1990) which allows inputs to overlap if they are close in the input space. The multi-resolution aspect comes from receptive fields being organized in this overlapping way.

The use of coding at several different resolutions has also been shown to speed up reinforcement learning with as much as an order of magnitude or more (Balkenius 1996; Sutton 1996). Features at low resolution are first associated with the rewarded responses, and the finer scales are subsequently used to fine-tune the behavior to specific input patterns. The multi-resolution representation promotes fast generalization while still supporting fine grained discrimination, by simultaneously coding for multiple similarities and differences.

Navon (1977) investigated multi-resolved perception in human subjects. Participants were shown pictures that could be interpreted both from a global and a local perspective, e.g., a large character made out of smaller ones. In one particular experiment, participants were required to judge whether such images were the same or different. The images were only shown for a brief time and could differ either at the local or the global level. Navon found that global differences were detected more frequently than were local ones.

Similarly, Schyns and Oliva (1994) studied how scenes are perceived in very fast recognition tasks. In two experiments, they investigated the roles of coarse and fine information when attempting to categorize natural scenes. Their results indicate that coarse processing is done first, which allows the visual system to form a rough approximation. This can be used to quickly classify what the overall scene represents. With time, more detail is added which can in turn be used to update the representation, or to refute the initial, raw estimate. On the other hand, Pomerantz (1983) explored how the perception of local parts compares with perception of wholes comprising those parts. They conclude that global precedence is not necessarily given, but depends on discriminability of the global level compared to the local one.

Given the effectiveness and usefulness of processing at multiple scales, it appears reasonable to assume that evolution has incorporated this strategy into biological perceptual mechanisms. Indeed, it is well known that the visual cortex codes information at different resolutions (De Valois et al. 1982; Ullman et al. 2002). According to the efficient coding hypothesis, the role of the early visual system is to produce an efficient representation for the visual input signal (Simoncelli 2003). If this is the case, we would expect cells in the visual system to respond to the relevant variation in natural images.

This line of inquiry has been explored by Bonin et al. (2011). They used high-speed calcium imaging to investigate the layout of the receptive fields in the primary visual cortex of rats. Random wavelet stimuli were used to allow the receptive fields of the neurons to be reconstructed from the calcium responses. The results show a global retinotopic shift in receptive-field position, but also a local scattering of cells with different receptive-field properties. Although not discussed by the authors, their results show considerable local variation in scale as well as orientation. They do not report finding any receptive fields for more complex patterns, but that may be due to their excluding patterns that explained less than 10% of the variation.

Van den Bergh et al. (2010) found a considerable variability on spatial tuning of cells in V1 and V2L in mice spanning almost two orders of magnitude, although most of the cells were tuned to approximately 0.1 cycles per degree. Similar results were found for V1 and V2 of macaque monkeys.

Interestingly, sensitivity to spatial frequencies has also been found in higher regions involved in scene perception, including the parahippocampal place area, the retrosplenial cortex, and the occipital place area (Kauffmann et al. 2015).

### Reverse hierarchy theory and the function of multi-resolved representation

Reverse hierarchy theory (Hochstein and Ahissar 2002; Ahissar and Hochstein 2004) proposes that top-down processes are required for increasing detail in visual perception, while bottom-up pathways principally mediate coarse and holistic representations. Furthermore, since information available to conscious awareness comes from the top of the perceptual hierarchy, top-down processes also start from this level, and work their way downward toward simpler, but more detailed representations. Bouvet et al. (2011) indicate that this principle holds not only for the visual pathways, but also for the auditory ones. Connected to reverse hierarchy theory, Campana et al. (2016) hypothesized that if the visual hierarchy constrains conscious vision by means of its structure, global orientations encoded at high levels of the visual hierarchy should be reported quicker than local orientations. They further hypothesized that if global orientations are prioritized, they might bias reports of local orientations.

They performed experiments with novel texture patterns designed to better distinguish local vs global processing than used by Navon (1977). Instead of Navon’s letters, they used circular textures made of oriented lines, and presented on a uniform gray background. A central rectangular area displayed aligned lines, but was surrounded by lines of random orientation. Among other things, they recorded magnetoencephalographic (MET) signals from participants while they were reporting orientation of local and global features. To get a measure of whether local or global features were most salient to subjects, they had them spontaneously report the orientation of stimuli, but without specifying which of the two levels to use.

Their findings support the initial hypotheses, although the difference in reaction time between global and local processing is in the order of 0.05s. Global orientation was also found to interfere with local orientation. Results from the MET experiment indicate that global information processing precedes local processing. This occurs even if global information is not task-relevant, suggesting it is automatic. This concurs with reverse hierarchy theory.

According to Ahissar and Hochstein (1997), multi-resolved receptive fields can be used for tasks with different complexities. For visual tasks, their findings indicate that learning of easier tasks uses more generalizations, but as tasks get more difficult, learning becomes more specialized. This maps to patterns of receptive-field selectivity along the visual pathway. Easy conditions generalize across position and orientation, while more difficult tasks narrow learning to particular orientations and positions.

### Anti-Hebbian learning and sparse coding

As stated above, reverse hierarchy theory of perception states that top-down processing begins at the top of the hierarchy, and with the most general representations (Hochstein and Ahissar 2002).

This implies that both coarse and fine representations exist in the perceptual hierarchy, but that the fine representations are not automatically activated. More detailed representations are implicitly more specific to stimulus. Hence, their activation should be more sparse. That is, when one population becomes active, others should be inhibited.

Anti-Hebbian learning and sparse coding (Földiák 1990) is a candidate for the mechanism behind this aspect of multi-resolution and reverse hierarchy theory.

Földiák (1990) proposed that inhibitive or anti-Hebbian connections in a neural population produce sparsely coded activation. That is, neural units tend to represent differences rather than similarities, and frequent patterns are represented by fewer units than infrequent ones. As the name implies, anti-Hebbian mechanisms produce decorrelated activation: Joint activity becomes less likely with time. This is in contrast with Hebbian-like association.

The advantages of sparse coding have been described by Olshausen and Field (2004), Földiák and Young (1995) and Barlow and Toraldo (1995). It allows multiple objects to be represented with little overlap and hence minimal ambiguity. At the same time, learning can be quick, since a given active population only need to represent a small number of patterns, and hence do not need extensive exposure.

### The mammalian canonical microcircuit

Given that the brain does indeed use multi-resolved receptive fields mediated by sparsely activated neural populations, and that anti-Hebbian learning via inhibitive inter-neurons is the mechanism by which it is achieved, the questions still remain where in the brain these processes take place.

Bastos et al. (2012) describe the canonical microcircuit, repeated across cortical columns of the mammalian visual cortex. The cortical column is divided into six layers, consisting of varying densities of pyramidal neurons and inter-neurons. Forward-projecting inputs to this circuit can be found in layer 4, outputs in layer 5 and 6, while layer 1 consists solely of inhibitory inter-neurons. Backward-projecting inputs from more frontal regions, are found in layers 1, 2, 3, as well as 5 and 6.

Hence, canonical microcircuits appear to have in place the required machinery for realizing multi-resolution and acuity, as well as top-down attentive processes that utilize them.

## Methods

To test how multi-resolution coding could develop in the cortex, we have designed a computational model which consists of a set of hierarchically organized layers of multi-resolution networks (MRNET). The model was implemented in the Ikaros framework (Balkenius et al. 2010) and trained on sequences of images to see how the receptive fields of the system would develop over time.

### Architecture

**Fig. 2:**
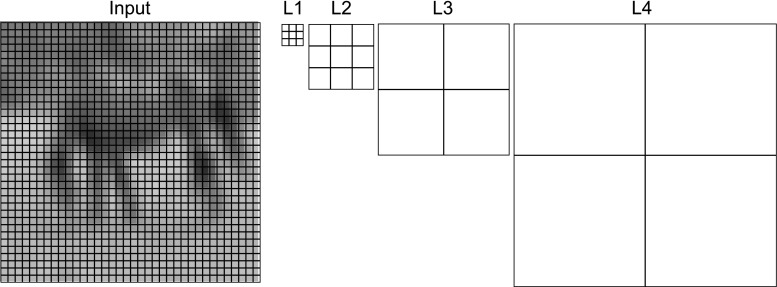
Size of receptive fields for layers 1 to 4, relative to 36 × 36 pixel input. Here, layer 1 consists of a 3 × 3 receptive field, with layer 2 being made up of a 3 × 3 grid of these, covering 9 × 9 pixels. Layer 3 doubles this, covering 18 × 18 pixels, which is doubled again in layer 4 to cover the whole 36 × 36 pixel image. The first layer uses overlapping fields with stride 1. Subsequent layers use dilated sliding window dot product (convolution) (Yu and Koltun 2015) to maintain spatial relationship with input. For example, the second layer covers three of the first layers receptive fields. But since the first layer uses overlapping fields, the second layer must have its receptive fields dilated to skip two neighboring fields. This is done to keep the sizes of the receptive fields as small as possible while still allowing them to cover large parts of the input

Figure 1 shows the basic computational architecture of the regeneration experiment. The input module reads a bitmap image from disk and transforms it into a tensor of real values. All MRNET modules have the same design, but are parameterized with the values shown in Table 1. The sizes of the receptive fields relative to the input are shown in Fig. 2. The output of a layer is equal to the activation of the neural units of that layer. The activation is given by the dot product of the input and the unit weights (see Eq. 2).

As shown in Fig. 1, the computational graph used for the current work is a simple four-layer system, where two 36 × 36 pixel gray-scale images are fed into layer 1, which uses filters of size 3 × 3 pixels, a stride of 1 pixel, and has a filter bank of 3 rows and 12 columns. The output of this layer is two matrices of 102 × 408 floating point numbers, one for each input image. The output tensor can be thought of as an array of 34 × 34 “cells”, each having a receptive field corresponding to a particular 3 × 3 patch of the input. Each such cell consists of a sub-tensor of dimensions 3 × 12, the same dimensions as the filter bank. This means that for each cell, the activity of each filter in the filter bank is given. The reason for using two-dimensional matrices like this is constraints of the Ikaros framework.

The above-described pattern is replicated for layers 2 to 4, but with one difference. Since receptive fields are overlapping in layer 1, and placed side by side in the output tensor, receptive fields in subsequent layers must be dilated (Yu and Koltun 2015; Wang et al. 2017), and use a stride equal to the size of the filter bank in the previous layer. For example, for layer 2, the receptive field should cover 3 × 3 of layer-1 receptive fields. Layer 2 receptive fields must then have the size of 3 × 3, 3 × 12, or 9, 36. Furthermore, each 3 × 12 block must be dilated or separated by 2 cells in the layer-1 output, which comes out as 2 × 3, 2 × 12, or 6 × 24 elements in the output tensor. The rationale behind using dilation like this is to conserve computational resources and to more easily relate receptive fields to the image inputs.

The output of the layer 4 module was fed back to an input which uses the learned weights to reconstruct an approximation of the input. This approximation is further piped through all the layers until a complete image is produced at layer 1.

In the current implementation, each layer in the MRNET allows an arbitrary number of bottom-up and top-down inputs. The number of each is independent. This allows learning from several streams, and also allows synthesis of multiple streams. The rationale behind this is first to allow sharing of the filter bank among streams and hence not having to re-train or use a separate network for different streams. Secondly, in cases where the required number of bottom-up and top-down streams is not equal, it saves computational resources. For example, in a system with an attentional component, it allows the center of attention to be one stream, while the context can be another; the choice can be made to only regenerate the center of attention, while using the context only for potentially selecting sets of actions or controlling other context-sensitive processing.

The streams in both directions are organized such that each are cycled through for the various operations done within the layer, such as rearranging the input tensor so that every receptive-field patch becomes a row vector; updating the filter bank; multiplying the input tensor with the filter bank to get the activity tensor; and rearranging the internal activity tensor into two-dimensional form to get the output tensor. Similarly for the top-down streams, they are cycled through when rearranging the top-down input; multiplying the top-down tensor with the filter bank; and decoding the top-down reconstruction tensor to get the top-down output by summing up overlapping receptive-field patches.

For the experiment conducted here, layers were set up to accept two parallel upstream inputs and one downstream reconstruction input. This allowed the layers to learn from both upstream inputs while reconstructing only the static image seen in Fig. 6. Using dot product activation with unsupervised learning has been demonstrated by Dozono et al. (2016), while Liu et al. (2015) show how self-organizing maps can be arranged in a hierarchical structure, though without using dot product activation and weight sharing. The work presented here differs from these previous works primarily in how weights are updated by means of cumulative inhibition. This will be explained in detail below.

### The multi-resolution network

**Fig. 3:**
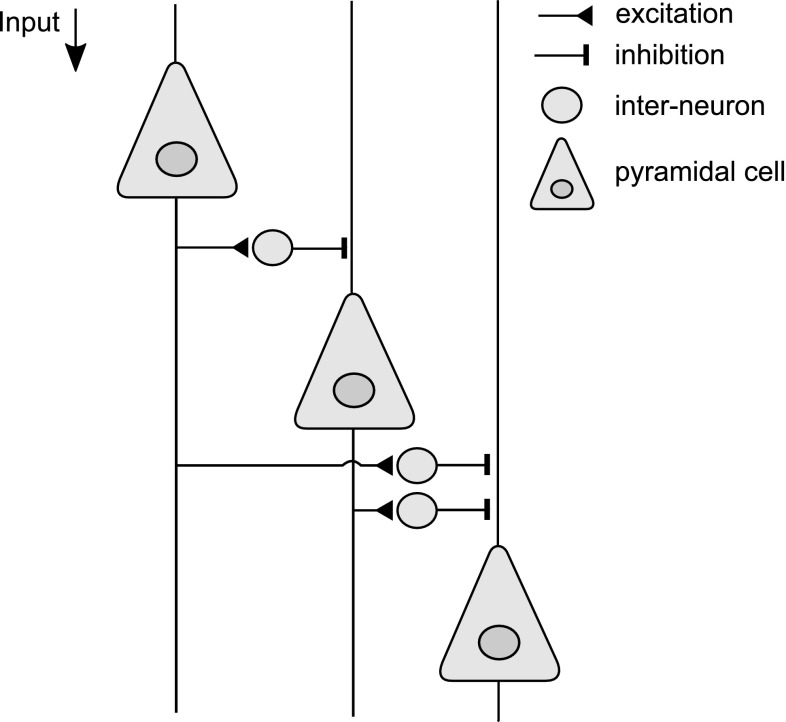
Pyramidal cells and inhibitive inter-neurons exerting cumulative inhibition in the cortical microcolumn of the visual cortex. The leftmost pyramidal cell here represents populations at the dense end of the sparsity gradient, and which respond to more general stimuli, while the rightmost pyramidal cell represents populations at the sparse end of the gradient. These respond to more particular, detailed stimuli

The central idea of the multi-resolution network is to add an unsupervised component to the location invariance of convolutional neural networks (LeCun et al. 1989). Additionally, the weight update algorithm uses cumulative inhibition of higher-order features to force weights to represent increasingly higher detail. The hypothesis behind cumulative inhibition is that pyramidal cells in visual cortex are differently affected by mutual inhibition, and thus that some cells experience heavy inhibition, while others less so. This should result in a gradient of inhibitive pressure across pyramidal cell populations. Since an inhibited cell is less likely to respond to the patterns that its inhibiting cells respond to, our hypothesis is that highly inhibited cells are responsive to significantly more diverse, and hence more detailed patterns than are less inhibited cells. Figure 3 shows a simple schematic representation for how this might work, with one pyramidal cell inhibiting two others, a second receiving inhibition from one, and inhibiting a third, and the third cell receiving inhibition by two others, but inhibiting none.

Each layer of the MRNET has a filter bank. This is a two-dimensional array of filter kernels, associated with an inhibition topology. In this work, all layers have the same filter bank topology, given by the function:

1 $$\begin{aligned} f((j_0, i_0), (j_1, i_1))= {\left\{ \begin{array}{ll} 1,&{} \text {if } (j_0 \le j_1 \text { OR } (j_0 = j_1 \text { AND } i_0 \le i_1))\\ 0, &{} \text {otherwise} \end{array}\right. } \end{aligned}$$

where $j_0$ and $j_1$ are row indices and $i_0$ and $i_1$ are column indices in the filter bank array. The function can be interpreted as the kernel at position $(j_0, i_0)$ inhibiting kernel at $(j_1, i_1)$.

The bottom-up activation $A\in {\mathbb {R}}^{n\times j}$ is achieved by multiplying the bottom-up input tensor $I\in {\mathbb {R}}^{n\times i}$ with the filter bank tensor $W\in {\mathbb {R}}^{j\times i}$ using standard matrix multiplication to get the dot product activation for each filter:

2 $$\begin{aligned} A = I W^T \end{aligned}$$

with $i\in \{3\times 3, 9\times 36, 20\times 40, 24\times 48\}$ and $n\in \{34\times 34, 28\times 28, 22\times 22, 10\times 10\}$ and $j\in \{3\times 12, 10\times 20, 12\times 24, 8\times 16\}$. Here, *n*, *i*, and *j* are ordered by layer. *I* is constructed by converting patches into rows using function *im*2*row* on the input, a row-based version of *im*2*col* (Function reference: im2col 2018b).

Similarly, since all operations involved in activation and weight update are linear, a MRNET can be used to generate images in a top-down fashion. This is done by multiplying the top-down input tensor $I_{TD}\in {\mathbb {R}}^{n\times j}$ with the filter bank tensor *W*, to yield $TD\in {\mathbb {R}}^{n \times i}$:

3 $$\begin{aligned} TD = I_{TD} W \end{aligned}$$

with *W*, *n*, *i*, *j* as given above. Rows of *TD* are converted to patches and assembled by a sliding version of function *row*2*im*, a row-based version of *col*2*im* (Function reference: col2im 2018a).

A central feature of the MRNET is the *inhibition buffer*, which models the influence on pyramidal neurons by inhibitory inter-neurons. The inhibition exerted on a given level is the cumulation of inhibition exerted on previous levels, as illustrated in Fig. 3.

**Fig. 4:**
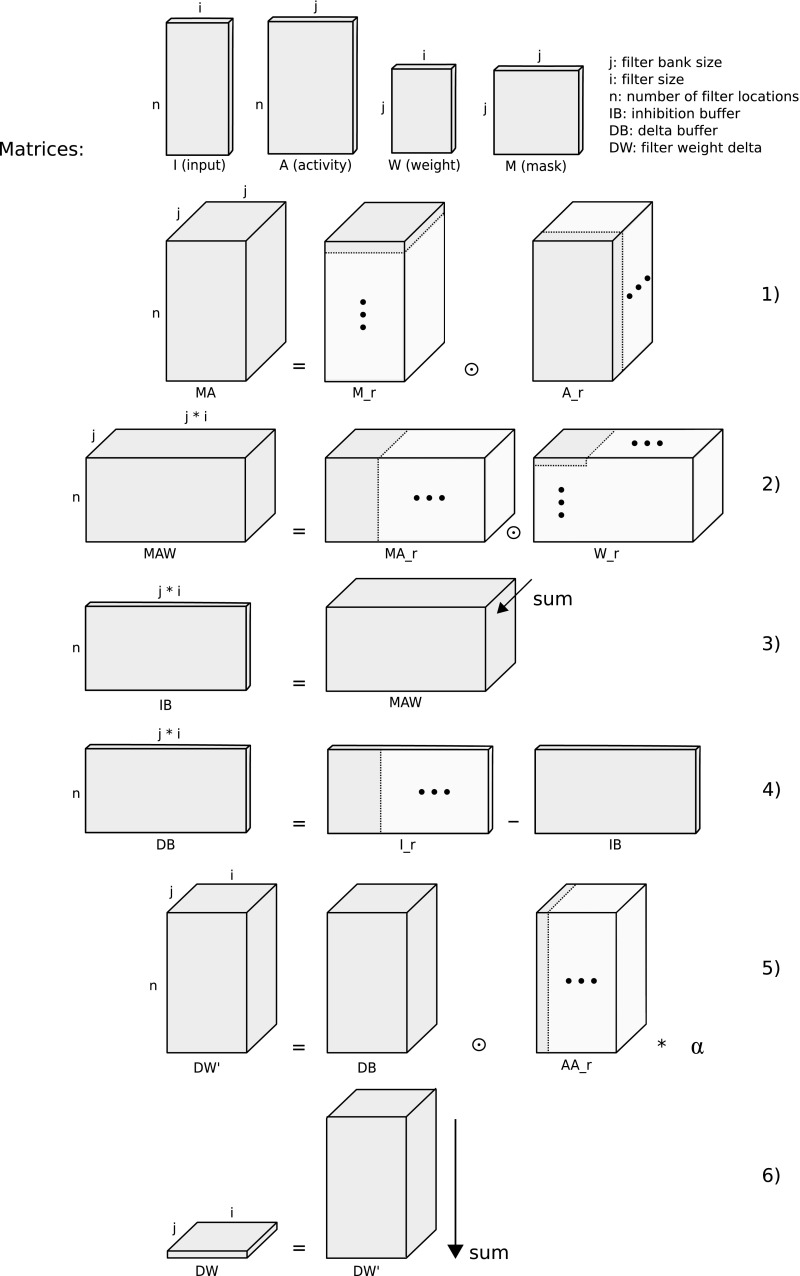
Illustration of weight update calculation. Operation (1) corresponds to Eq. 4, and shows the mask tensor *M* and activity tensor *A* being tiled into tensors $M_r$ and $A_r$, then multiplied per element to yield tensor *MA*. Operation (2) corresponds to Eq. 5, and shows tensor *MA* being tiled in the x dimension, yielding tensor $MA_r$, then being multiplied with tensor $W_r$, which is the result of tensor *W* being tiled along dimensions x and y. The per element multiplication yields tensor *MAW*. Operation (3) corresponds to Eq. 6, showing how tensor *MAW* is summed along the z dimension to yield inhibition buffer tensor *IB*. Operation (4) corresponds to Eq. 7 and shows how tensor *I* is tiled along the x dimension to yield tensor $I_r$, from which tensor *IB* is subtracted, to yield the delta-buffer tensor *DB*. Operation (5), corresponding to Eq. 8, shows then how the activity tensor *A* is replicated along dimension x to yield tensor $AA_r$, and multiplied per element with tensor *DB* and the learning rate $\alpha$, yielding intermediate tensor $DW'$. Finally, operation (6), which corresponds to Eq. 9, shows how tensor $DW'$ is summed along the y dimension to yield the weight update tensor *DW*

The inhibition buffer is implemented in several steps, involving the tensors *I*, *A*, *W* given above, as well as the inhibition mask $M\in {\mathbb {R}}^{j\times j}$. See Fig. 4 for a graphical overview of the tensors involved, and the weight update process.

The process of calculating the inhibition buffer is initiated by setting up the inhibition mask *M*. This is done by iterating over each element in the mask and applying the topology rule of choice. See Eq. 1 for the topology rule used here.

The next step is to apply the mask to the activity tensor. This is done by tiling both the inhibition mask and the activity tensor into $M_r, A_r \in {\mathbb {R}}^{j \times n \times i}$ and multiplying by element:

4 $$\begin{aligned} MA = M_r \odot A_r \end{aligned}$$

Tiling operations are done to allow the use of efficient tensor operations and thus to conserve computational resources.

*MA* and *W* are again tiled yielding tensors $MA_r, W_r \in {\mathbb {R}}^{j \times n \times ji}$. These are multiplied by element:

5 $$\begin{aligned} MAW = MA_r \odot W_r \end{aligned}$$

The inhibition buffer is then obtained from the product *MAW* by a summing dimensional reduction along the first dimension, resulting in the inhibition buffer tensor $IB\in {\mathbb {R}}^{n \times ji}$. Given elements $ib_{n, ji} \in IB$, and elements $maw_{n, j, ji} \in MAW$:

6 $$\begin{aligned} ib_{n, ji} = \sum _{c=1}^j maw_{c, n, ji} \end{aligned}$$

with *n*, *j*, and *i* as given above.

Next, the bottom-up input *I* is tiled to a tensor $I_r\in {\mathbb {R}}^{n \times ji}$. A temporary delta-buffer tensor *DB* is calculated by subtracting the inhibition buffer *IB* from the tiled input tensor:

7 $$\begin{aligned} DB = I_r - IB \end{aligned}$$

The delta buffer is resized, and the activity tensor *A* is tiled to yield $DB, AA_r\in {\mathbb {R}}^{j \times n \times i}$. A temporary tensor $DW'$ is then obtained by multiplying the delta buffer by element with the tiled activity tensor $AA_r$ and the scalar learning rate $\alpha$:

8 $$\begin{aligned} DW' = DB \odot AA_r * \alpha \end{aligned}$$

The final weight delta is obtained by summing elements $dw'_{n, j, i}\in DW'$ along the first dimension to yield a tensor with elements $dw_{j, i}\in DW|DW\in {\mathbb {R}}^{j \times i}$:

9 $$\begin{aligned} dw_{j, i} = \sum _{c=1}^n dw'_{c, j, i} \end{aligned}$$

Tensor *DW* can be then be added to the filter bank directly:

10 $$\begin{aligned} W_{t+1} = W_t + DW \end{aligned}$$

**Table 1:** Hyper-parameters for MRNET layers 1–4, given in a row and column format

	Layer 1	Layer 2	Layer 3	Layer 4
Receptive field	3, 3	3, 3	2, 2	2, 2
Filters	3, 12	10, 20	12, 24	8, 16
Learning rate	0.0005	0.0005	0.0005	0.0005

### Training

Training was done layer-wise, starting with layer 1, on the CIFAR-10 dataset (Krizhevsky and Hinton 2009) until the smoothed absolute value of the derivative of the difference between the input and the reconstruction was less than a threshold value (1e−4). The rationale behind this somewhat complicated stopping criterion is that the optimum stopping point in time was unknown, as was the degree of reconstruction similarity that could be achieved.

**Fig. 5:**
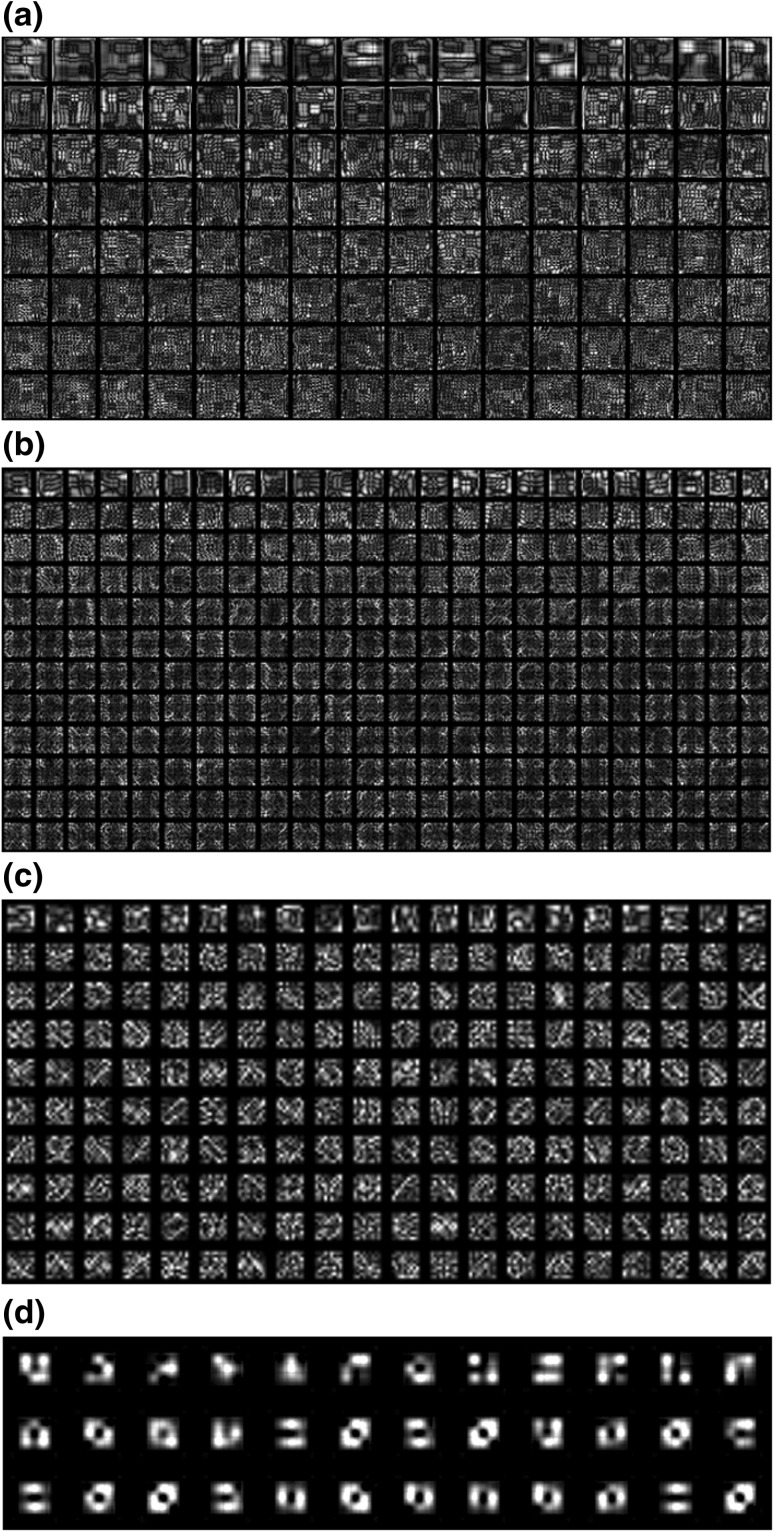
The image of layer 4 weights shows the learned filters, covering the whole image, or 36 by 36 pixels. Close scrutiny of the top row reveals coarse patterns made up of finer ones. **a** The shapes are characteristic of the cumulative inhibition algorithm, with broader and lighter patches limited by thinner darker strips. **b** 18 by 18 receptive field filters in layer 3. **c** 9 by 9 receptive field filters in layer 2. **d** 3 by 3 receptive field filters in layer 1

Images of the weight filters (Fig. 5) were produced by activating each of the filters by turn and regenerating the receptive field in a top-down fashion.

As will be elaborated on below, the presented architecture potentially opens up intriguing possibilities for unsupervised learning of visual building blocks that might be used for robotic applications like obstacle avoidance. Furthermore, a system using a MRNET might be used to model aspects of visual abstraction by inhibiting high detail filters. The top–down constructive algorithm might be used to elucidate such cognitive processes as imagination and creativity.

## Results

**Fig. 6:**
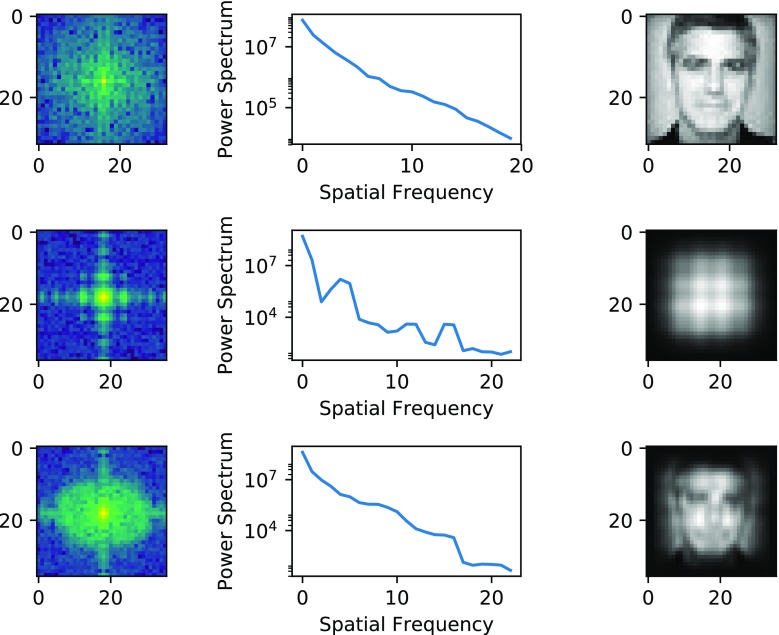
Frequency spectrum for input image (top), with top–down reconstruction before training (middle), and after training (bottom). It can be seen that the input image is near log linear across spatial frequencies. Artifacts due to the reconstruction process results in false peaks at about 3, 11, and 15 Hz in the reconstructed image before training. As training is completed, power increases across the spectrum in the reconstructed image, but power is cut off at about 18 Hz. Reconstruction is limited at the upper end of the frequency scale by the size of layer-1 receptive fields

**Fig. 7:**
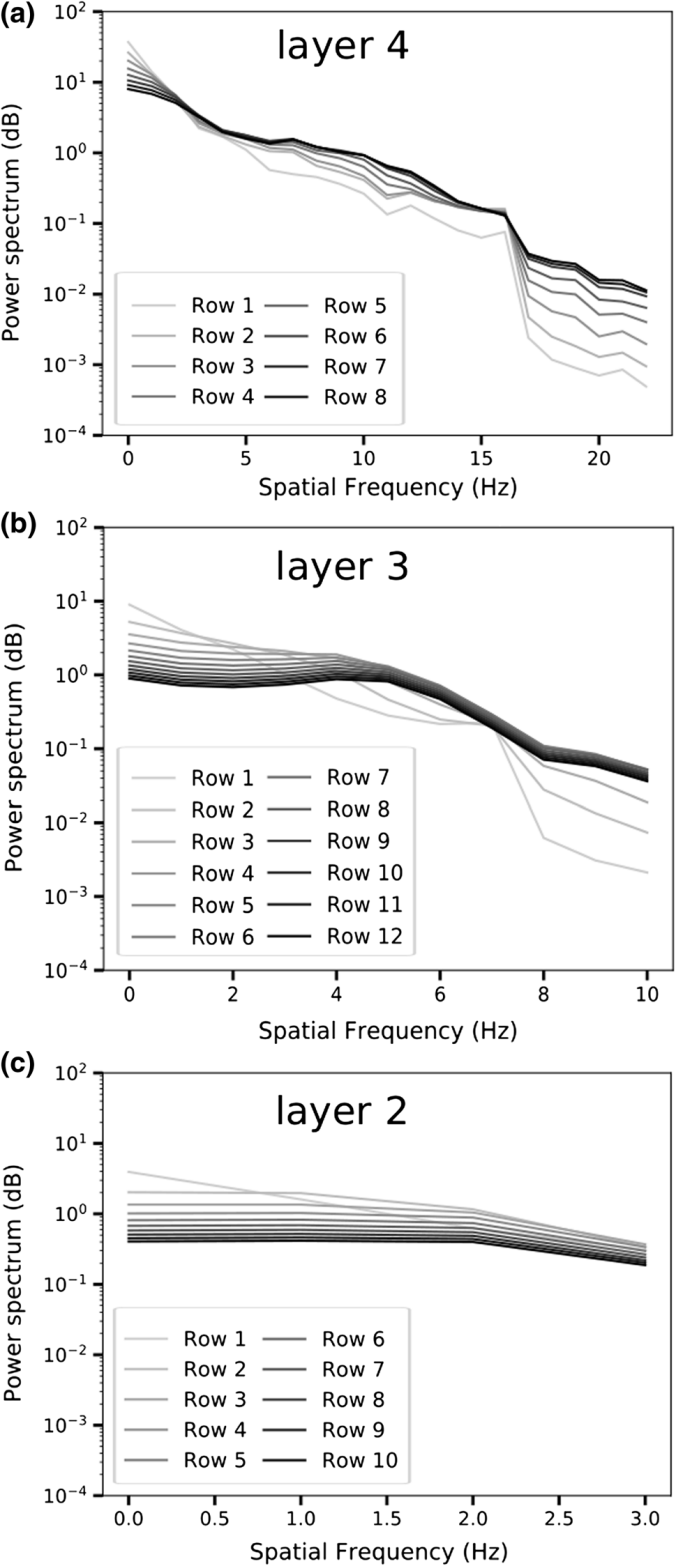
Average of power spectrum of rows of weights for layers 2–4. Rows correspond here to the rows of receptive fields shown in Fig. 5, counting from the top down. Spatial frequency scale is relative to the size of receptive field, such that layer-2 frequencies should be read as being at the rightmost end of the layer 3 and 4 scales. **a** Layer-4 plots indicate that the fields are not uniformly responsive across spatial frequencies, and that they converge at two points: at around 2Hz, and around 16Hz. The plots reveals also that there is little difference in power response for rows 6, 7, and 8, indicating that the latter two may be omitted to reduce computational overhead without much affecting reconstruction quality, or the information encoded by the layer. **b** Layer 3 covers 2 × 2 of layer 2 receptive field, or 18 × 18 pixels of input image. Rows 1 to 3 have clearly differentiable power responses, which converge at around 7 Hz. Rows 4 to 12 have very similar response after about 5 Hz, indicating that rows 5 to 12 may be omitted to reduce computational overhead. **c** Layer 2 covers 3 × 3 of layer-1 receptive fields, corresponding to 9 × 9 pixels of input image. Row 1 has the highest power response up to about 0.6 Hz, but declines from the onset. Row 2 has a flat response up to around 1Hz, where a knee appears. A second knee appears around 2 Hz. Subsequent rows are flat up to around 2 Hz then declines. Rows 4 to 10 have a power response of 1dB or less across the frequency spectrum

**Fig. 8:**
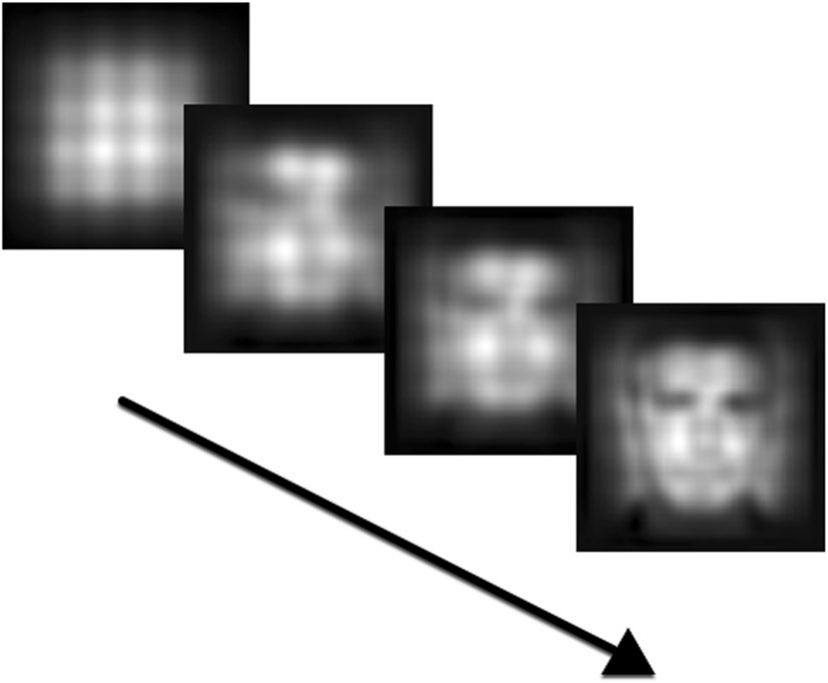
Increasing detail of regenerated image at iterations 2, 50, 200, and 800, as layer 4 stabilizes. Square pattern and darkened frame are artifacts of the regeneration process, and formatting of receptive fields. First, dark patches around the eyes are formed, at iteration 2. At iteration 50, the contours of the face become visible. Most of the coarse detail is present at iteration 800; the contours of the face are salient, and the nose and mouth can be made out

**Fig. 9:**
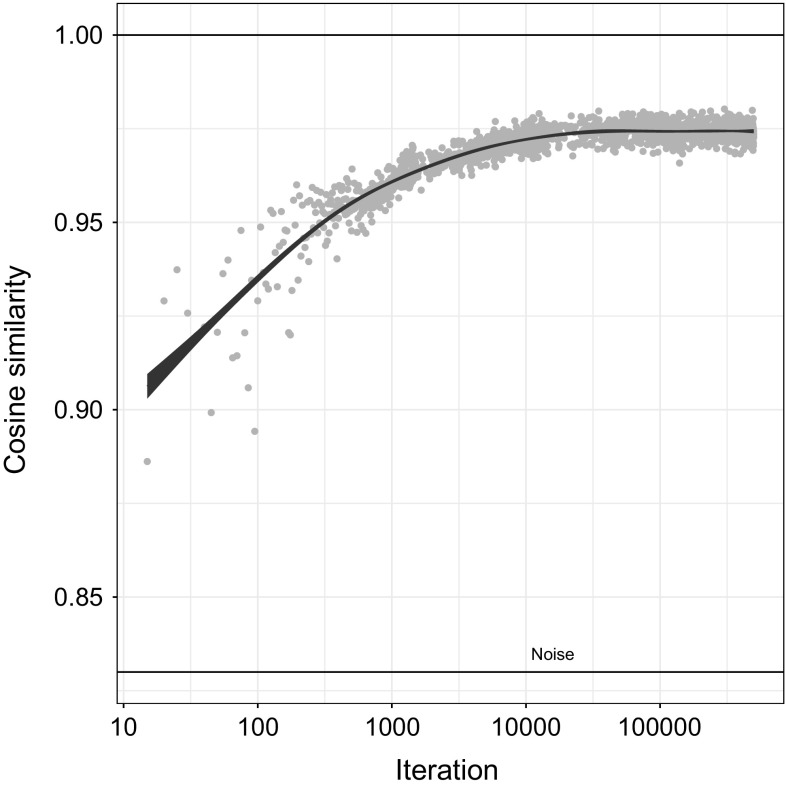
Change in cosine similarity over time between input and reconstructed image, with fitted smoothing function and noise floor. Dataset has been thinned from 100,000 to 1800 data points. Logarithmic scale for iteration pushes most data points toward right-hand side of plot, highlighting the variance in similarity the first 300 iterations. From iteration 1000 to 30,000 similarity levels off, eventually reaching about 0.97

Figure 5 shows reconstructed weights for layers 1–4, where layer 1 is closest to the input. For each layer, the topmost row is at the coarse end of the coarse-fine spectrum, with the bottom-most row representing the finest detail.

As shown in Fig. 6, frequency content of the reconstructed image consists mostly of low frequencies before training, but is much closer to the original after training. There is a noticeable dip above 15Hz in the trained image, and also one below 5Hz. Figure 7 shows average frequency content of weights in each row of layer 4. As can be observed, there is a clear progression, where the low-frequency power attenuates, while high-frequency power increases. There is also a noticeable increase in power in middle frequencies. The difference is small, however, for row 7 and 8.

Figure 8 indicates that layer 4 is able to represent an appreciable portion of the information of an input image by 1000 iterations. Though it still might lack enough detail for determining who the picture represents, there is qualitatively enough for category identification.

The input image and the reconstructed images were normalized and cropped to remove dark framing artifacts. The cosine similarity was then calculated at every fifth iteration. Figure 9 shows how the similarity increases the first 10,000 iterations, then begins to taper off from iteration 30,000.

To the extent that the results above support that the MRNET can represent input in a coarse-to-fine manner, there are several cases in which it might be of use. Starting with the bottom-up pathways, the MRNET affords unsupervised learning of visual building blocks. This means that the network can be trained on unlabeled data. In a robotic system, it would suffice for the robot to be active and observing the world for the network to begin learning.

The building blocks can also be used before the system has fully stabilized and can optimally represent input. A coarse outline of the world can be useful for such things as obstacle avoidance, and for beginning exploration. As Fig. 8 shows, the outline of a face takes only a couple of thousand of iterations to be formed, when preceding layers have stabilized. As a first approximation, this might be enough to distinguish people from other things.

Related to this, the approximations yielded by the top rows of the filter banks may be used for fast recognition of particular important categories. In humans and monkeys, such unrefined patterns appear to be used for avoiding dangers in the natural environment, such as snakes and spiders (Globisch et al. 1999; LoBue and DeLoache 2008; Shibasaki and Kawai 2009). For a household robot, analogously important categories might be staircases, people, or pets like dogs.

Moving into the more traditionally cognitive domain, a coarse-to-fine representation might prove to be highly useful as a mechanism for abstraction. Selectively attenuating the filters representing detail yields a more abstract representation of the input. Such representation might then be used for comparisons, enabling inputs that would otherwise be judged different to be judged same.

Depending on how the filter bank of the layers is configured, the MRNET can be used to compress input representations while retaining important features. The specific requirements of the application dictate how much detail is required, and hence how many rows and columns of the filter bank should be used. As is indicated by Fig. 7, there appears to be a limit to the number of filter bank rows that contain useful information. Hence for a given input resolution, it should be possible to determine by means of FFT analysis which configuration gives the required level of detail.

Since the MRNET affords linear combination of weights in a top-down fashion, it can be used as a component of a generative model. By connecting the topmost layer to a suitable probability distribution, samples can be produced within a target domain of choice.

Related to this, the MRNET can function as the generative end of a model of visual imagination. Such a system would likely require the MRNET to be connected to some form of sequential memory, i.e., a model of hippocampus (Hassabis and Maguire 2007; Hassabis et al. 2007; Balkenius et al. 2018), as well as a network storing object-related information to bias the MRNET toward producing meaningful combinations.

By extending such a model of visual imagination to other sensory modalities, such as sound, and adding an associative network between them, as well as an evaluative component based on the human reward system (Runco and Chand 1994, 1995), it is plausible that interesting models of creativity could be constructed. Here, the associative network would have the role of providing associations with low probability, i.e., novel associations, while the reward system would be required to evaluate the usefulness, interest, or intrinsic value of those association.

## Discussion

**Fig. 10:**
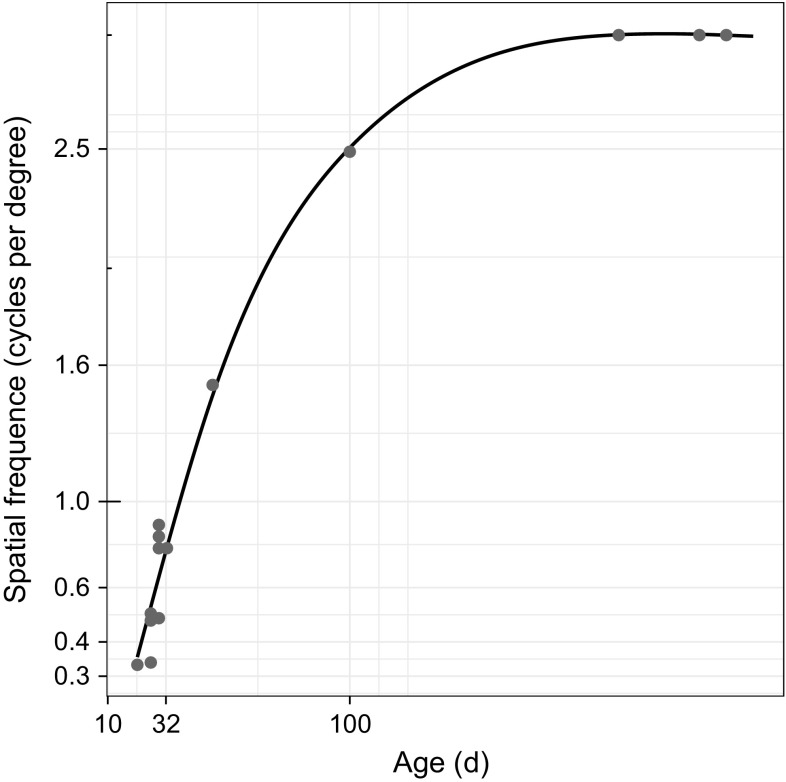
Plot of developing visual acuity in kittens, reconstructed from Freeman and Marg (1975), with fitted smoothing function

We have shown that a hierarchy of MRNET neural network layers can adapt to the statistics of input signals, such that those signals may be approximately regenerated. Furthermore, representations are learned in a coarse-to-fine manner with separate receptive fields for different levels of detail.

In this section, we discuss different aspects of cumulative inhibition. We start with comparing the performance of our algorithm with biological data from the perspective of the development of acuity. Next, we discuss how cumulative inhibition might be realized in the cortical microcircuit, and evidence supporting this notion. Then, the concept of multi-resolution is discussed, and how the MRNET algorithm can be thought of as a transformation of input data into multi-resolved space. Following this, we compare our model to other computational models of coarse-to-fine processing. We then address some criticisms against sparse coding and suggest how they might be resolved in the context of cumulative inhibition. Lastly, we discuss limitations of the current model, and make suggestions for further work.

Although the measure of cosine similarity between a reconstructed image and the input it is constructed from as seen in Fig. 9 cannot be directly compared to a measure of neural response to sinusoidal grating stimuli shown in Fig. 10, there is nevertheless intriguing similarities between the two. Firstly, the argument can be made that the two measures are somewhat symmetrical, in that the former shows an increasing similarity between a detailed input and an increasingly detailed reconstruction, while the latter shows an increasing sensitivity to detailed input.

Comparison with the results of Freeman and Marg (1975) highlights some of the differences between our model and measurements of biological systems. Figure 10 shows how acuity increases rapidly the first 100 days and then plateaus within the first year or so. The time scale of measurements in our experiment, and shown in Fig. 9, is limited to 500,000 iterations. The rapid adaptation is hence less salient in the model data, but the general shape of the curves is similar. Due to distribution of data points along a logarithmic time axis, the smoothed plot shows more variability in the first 300 iterations. The time scale for the model is shorter than that of Freeman and Marg (1975), but the number of data points is much higher.

The exact rate of adaptation that corresponds to the biological changes, however, remains to be determined.

The functionality of a single MRNET layer can be mapped loosely to that of the canonical microcircuit found in cortical columns of mammalian visual cortex (Bastos et al. 2012). Given that the role of some inter-neurons are to mediate shaping of receptive fields for perception of higher detail, as indicated by Huang et al. (1999), their role corresponds to the calculation and application of the MRNET inhibition buffer. Forward activation in a MRNET layer corresponds naturally to activity of pyramidal neurons.

The principle of cumulative inhibition suggests that pyramidal cells in visual cortex are heterogeneously inhibited, which would appear to implicate that signals carrying more detailed information should be weaker than those carrying less detailed information. However, Xue et al. (2014) indicate that inhibition is balanced with excitation in this area. One possible way of reconciling these two notions is that pyramidal neurons that represent high detail, and which are therefore the target of much inhibition, are also balanced by an equal amount of top-down excitation. Alternatively, neurons representing coarse detail may be attenuated by top-down inhibition.

Bastos et al. (2012) describe the canonical cortical microcircuit, which is also a building block of areas related to the visual pathway. Layer 2/3 receives backward projection from more frontal regions, and Wagatsuma et al. (2011) showed that some of these projections carry signals related to attention. Specifically, they tend to excite pyramidal cells in layer 2/3, but inhibit pyramidal cells in layer 5.

Binzegger et al. (2009) performed a topological analysis of cat V1 microcircuits and quantified the number of synapses for different cell types in the six layers. Intriguingly, they found that layer 2/3 pyramidal cells have the highest number of synapses, and also that inhibitory basket cells in these layers have the most effective synapses. Interpreting this from the perspective of cumulative inhibition, it could mean that attentional signals are required to overcome inhibitory pressure for cells that represent high detail. Another implication appears to be that attending to detail inhibits perception of overall structure. This would also fit well with reverse hierarchy theory (Hochstein and Ahissar 2002; Campana et al. 2016), which posits that automatic, bottom-up perception is coarse and holistic, and requires top-down attention to become more detailed and localized.

Although our model currently does not have support for top-down excitation and inhibition, it does allow for it. In the version of the MRNET weight update algorithm presented here, top-down signals are only used for generation.

It is currently unclear how the processes of self-organization of receptive fields relate to the formation of receptive fields that represent specific categories and patterns, but they might reflect different phases of maturation processes. It is likely that self-organization only happens during an organism’s critical period, while categorical sharpening can continue throughout the life span and hence reflects more plastic processes. These processes might also involve extra-occipital structures like hippocampus and parts of temporal cortex (Keresztes et al. 2017).

While a conventional convolutional network performs a (usually) nonlinear transformation of inputs into a space determined at the highest level by the number of weights at that level (Schmidhuber 2015), the MRNET forward activation transforms inputs linearly into a space that contains discrete information about level of detail. The accumulated information can be passed on to a classifier, but since the transformation is linear and not class specific, we found that the classifier performs no better than when fed raw image data.

Hence, the contribution of this work is not to improve classification, but to elucidate and improve understanding of the mechanisms involved in perception, and how they develop. Specifically, it suggests a way in which excitatory and inhibitory neural populations cooperate to adapt to sensory stimuli.

The results indicating that the output of a MRNET stack gives similar classification performance as raw image data predict that a self-organized process by itself is not sufficient to account for the discriminative abilities of mammals in general, and humans in particular. Rather, it is likely that a process of specialization is also involved, whereby some neural populations form receptive fields that are specific to particular salient stimuli. This would allow fast recognition of such stimuli, and also association of stimuli with specific behavior. Put another way, the self-organized process allows perception of all stimuli, but not necessarily recognition and categorization of those stimuli.

To elaborate, the receptive fields generated by the MRNET algorithm have a principal component-like nature, which means that they are used as building blocks to assemble a signal. Similar building blocks may thus be the outcome of processes taking place during the critical period of visual system development. Most inputs can be assembled from them, but they do not represent particular percepts. To represent specific categories, the building blocks must be put together in a particular way, and stored, perhaps, in a separate network.

Figure 5 shows that in layer 1, many weight patterns are repeated several times. It is also not obvious that the different rows of weights represent different level of detail. One reason for this is the small size of the receptive field. With only 3 by 3 pixels, the number of combinations is much more limited than in layers 2–4. It appears, though, that the dimensions of the weight matrix are not optimal, and that particularly the number of columns could be reduced to limit the number of very similar weight patterns.

The difference in detail level of the patterns is more easily seen in layer 4, where in particular the two topmost rows have coarser patterns than rows further down. By simple inspection of Fig. 7, it is hard to notice the increasing level of detail that the patterns represent, but by comparing each rows’ average power spectrum, there is nevertheless a visible trend, where lower frequencies are attenuated, and the highest frequencies steadily increase. This indicates that the mechanism of cumulative inhibition, whereby the inhibitive pressure increases with each additional row, does indeed force weights to represent ever finer detail.

The MRNET algorithm can be compared with the approaches of Földiák (1990), Witkin (1984), Viola and Jones (2001) and Sutton (1996). The common element between these is multi-resolution representation. Except for Földiák (1990), the difference between the MRNET algorithm and the above is unsupervised learning of feature vectors, and hence what kind of patterns those vectors represent. There is also a difference in how network units are activated. The MRNET units are activated using dot product computations, like in other types of convolutional networks (LeCun et al. 1998).

Witkin (1984) uses manually defined Gaussian filters with variable smoothing settings to achieve multi-resolution representations. The approach by Viola and Jones (2001), on the other hand, is to use rectangle representations, which are reminiscent of Haar basis functions (Haar 1910). That is, feature vectors are composed of adjacent high- and low-intensity rectangles oriented horizontally, vertically, or diagonally. Sutton (1996) achieves multi-resolution by using multiple overlapping tilings, each of which represents a smaller area of the input. Multi-resolution is here used in a reinforcement learning context, such that the tilings represent parts of a continuous state space, and are used to efficiently associate optimal action policies with areas of that space.

The MRNET learning algorithm is based on Földiák (1990), which proposed the concept of learning in the inhibitory connections in artificial neural networks. This network learns also in an unsupervised manner, and produces decorrelated receptive fields. This again ensures that the network is sparsely activated for a given input. But Földiák (1990) does not arrange inhibitory connections in such a way that they become cumulative and hence can represent a coarse-to-fine gradient. Thus, the MRNET algorithm can be viewed as an extension of Földiák (1990), achieved by changing the topology of the network with regards to inhibitory connections.

Spanne and Jörntell (2015) critique the concept of sparsity in neural simulation models, pointing out that empirical evidence for sparseness in biological systems is not strong, and furthermore that defining this property in a biological neural network is itself problematic. There are also indications that sparseness can decrease with experience and with wakefulness (Berkes et al. 2009). Their main argument against sparseness is, however, that it does not support generalization well. This criticism is valid; increasing sparsity implies less overlap between active sub-populations for a stimulus, which at the extreme means particular stimuli being represented by a single cell (Barlow 1972).

We propose that perceptual networks are neither homogeneously dense nor sparse, but that cumulative inhibition results in a dense-to-sparse gradient. With such a topology, generality is served by populations at the dense end of the gradient, while specificity is represented at the sparse end. The observation by Zylberberg and DeWeese (2013) that sparsity decreases with development is not reflected in our model, particularly not at higher layers. However, Zylberberg and DeWeese (2013) discuss mainly V1 simple cells. Such cells cover very small areas of the input fields and are usually thought to represent basic visual building blocks like edges (Hubel and Wiesel 1968). One interpretation is that receptive fields become with experience more diverse in terms of the edge angles they represent, but that as the angles grow less different, more cells are partially activated for a given input. Thus, the activation as a whole becomes less sparse.

Spanne and Jörntell (2015) pose the question of what is the role of inhibitory inter-neurons in perceptual networks. Our models suggest that one role might be the mediation of acuity and coarse-to-fine detail.

Some recent works have similarities with the work presented here. Trappenberg et al. (2015) show how a SOM can be augmented with back-propagation of errors and a hierarchical structure to improve clustering. The authors indicate that the overall accuracy of their system does not rival state-of-the-art deep learning architectures, but that their main intention is to elucidate how biological networks solve classification problems. They found that back-propagation errors help to improve the visual representation of the clustering, but does not significantly improve classification performance as such. Trappenberg et al. (2015) do not employ the practice of convolution kernels and weight sharing. Their use of back-propagation to modify weights also sets their work apart from ours.

Liu et al. (2015) use a hierarchy of layers consisting of arrays of local SOMs, representing parts of the image. Each of these layers are coupled with a sampling layer which for each SOM cell chooses only the winning unit. SOM layers and sampling layers are assembled to form a classifier that according to the authors outperform a supervised SOM architecture, but does not challenge state-of-the-art deep back-propagation systems. Although this work uses local representations akin to that of convolutional networks, the authors do not use weight sharing or dot product activation.

Dozono et al. (2016) show two versions of how convolution and pooling layers can be used with SOMs in a hierarchical configuration. A sliding window is used to learn kernels using the batch SOM algorithm. Correlation coefficients are then computed of input vs kernels in the convolution layer. This array of correlation coefficients is then sent to a pooling layer. In the first version, standard max pooling is used. The output of the pooling layer is then used as input to a SOM layer again. The second version adds a layer that calculates the euclidian distance between each of the winning nodes, then passes the resulting array to a pooling layer which computes the minimum of these distances. The output of this min pooling layer is then passed to the next SOM layer. Each of the models repeat the layer pattern of SOM and pooling three times.

Dozono et al. (2016) are closest to the model presented here. In particular, the coefficient correlation array corresponds to the bottom-up activation array used in the MRNET layers. The practice from convolutional networks of using a sliding window to update weights and use of weight sharing across the input is also similar. The main differences between the works cited above and what we present here are firstly in how weights are updated, using cumulative inhibition, and secondly the support for regeneration in our model.

The current version of our model has several limitations. The MRNET algorithms can transform input into a multi-resolved representation, but is limited in that this transformation is holistic. This means that there is no figure-ground separation. Additionally, since the learning is unsupervised, weights are not shaped to represent particular categories. Further processing is required to achieve good classification abilities. Lacking also are mechanisms for perceptual grouping, such as by size, color, and proximity. Hence, in a perceptual system with abilities similar to that of mammals, additional functionality would be required for object recognition.

In future work, we plan to focus on establishing optimal hyper-parameters for the MRNET network, particularly what the minimum dimensions of the weight map should be to achieve acceptable detail representation. Further research is also necessary to clarify how effective categorization and object recognition can be achieved using the multi-resolution output of a MRNET based network. Finally, determining whether top-down activation can be used fruitfully to modulate the upward stream would be a highly interesting path of inquiry.
